# Evolutionary Game Analysis of Construction Workers' Unsafe Behaviors Based on Incentive and Punishment Mechanisms

**DOI:** 10.3389/fpsyg.2022.907382

**Published:** 2022-05-24

**Authors:** Jianbo Zhu, Ce Zhang, Shuyi Wang, Jingfeng Yuan, Qiming Li

**Affiliations:** ^1^School of Civil Engineering, Southeast University, Nanjing, China; ^2^Department of Civil and Environmental Engineering, National University of Singapore, Singapore, Singapore

**Keywords:** construction workers, unsafe behavior, mechanism design, evolutionary game, incentive and penalty

## Abstract

Construction is one of the most dangerous industries because of its open working environment and risky construction conditions. In the process of construction, risk events cause great losses for owners and workers. Most of the risk events are closely related to unsafe behaviors of workers. Therefore, it is of great significance for contractors to establish management measures, e.g., incentive and punishment mechanism, to induce workers to reduce unsafe behaviors. This paper aims to take the incentive and punishment mechanism into consideration and develop an evolutionary game model to improve the effectiveness of safety management. The evolutionary stability strategies which can help reduce unsafe behaviors are obtained and analyzed. Results show that there are 12 equilibrium strategies under the condition of different parameters. Specifically, the incentive and punishment mechanism has played an important role for the evolution direction. A balanced incentive and punishment mechanism for the investment and positive stimulus for workers can effectively promote both sides to take positive behaviors, and then realize good evolutionary stable situations. In addition, the initial perceptions of both sides have a decisive impact on the evolution direction. Strengthening communication with the mutual trust between both sides can improve safety performance of both sides. This study is valuable for contractors to design appropriate incentive and punishment measures and establish relevant strategies to promote safe behaviors of construction workers.

## Introduction

The global construction industry has maintained a rapid growth in the past decades. Taking China as an example, the total output value of the construction industry has increased from 13.7 trillion RMB to 29.3 trillion RMB in the past decade (2012–2021), accounting for about 25% of the total GDP for a long time. Thus, the construction industry is a driving force of national economy. Compared with the manufacturing industry, the working environment of the construction industry is more open with complex construction site conditions. During the construction process, the main structure of the building is constantly changing with a large number of temporary construction facilities. These adverse factors contribute to on-site risks frequently (Moosa and Oriet, 2021). Therefore, safety management is a core management problem on site. The evidence from Hong Kong shows that 80% of industrial accidents occur in the construction field (Labor Department, 2021). According to data of China Ministry of Housing and Urban-rural Development, safety accidents related to the production of housing and municipal engineering led to 840 and 904 deaths in 2018 and 2019 respectively (Standardization of Engineering Construction., 2020). The safety situation in the construction industry is an on-going concern.

Various causes can lead to construction accidents, and unsafe behaviors of workers are considered to be one of the important sources (Guo et al., 2018; Inyeneobong and Boluwatife, 2022). Unsafe behaviors of workers mean that their behaviors deviate from safe procedures (Shin et al., 2014). Several factors contribute to unsafe behaviors (Su et al., 2019). For example, Fang et al. (2015) found that the accumulation of fatigue would significantly reduce workers' control abilities. Man et al. (2021) studied the influence mechanism of individual and organizational factors on risk-taking behaviors of workers. Jiang et al. (2015) identified the personal, environmental and organizational conditions which affected affect workers' behaviors through cognitive analysis and established a system dynamics model to explain how it affect these behaviors. Cheng et al. (2022) provided a systemic review of the application of electroencephalogram in computing construction workers' cognitive statuses which affect their safety and productivity. Xia et al. (2020) formed the antecedent model of worker's unsafe behaviors through the literature review of empirical research and the model included 83 factors which were divided into 5 groups.

It can be found from the accident records in construction industry that numerous safety accidents or accident symptoms have occurred due to mistakes or the negligence of managers (Teo et al., 2005; Huang and Hinze, 2006). Safety management is one of the most important factors to promote the implementation of safety measures and workers' occupational safety, which significantly affect unsafe behaviors. According to a survey of a construction for affordable housing in Nanjing which is located in eastern China, contractors set up safe construction awards regularly, and punished workers for unsafe behaviors, including smoking, not wearing hard hats, etc. Contractors were also actively exploring the use of closed circuit video equipment and artificial intelligence to reduce the cost of monitoring workers' unsafe behaviors and improve effectiveness. Many scholars have researched on safety management. Lu and Yang (2010) and Mearns and Reader (2008) found that safety policies and concerns could positively influence workers' behaviors by collecting survey statistics from workers. Jiang et al. (2014) concluded that punishment was the most effective method of correcting workers' mistakes by means of modeling workers' safe behaviors and testing the impact of incentive measures on workers' behaviors. Haas (2020) pointed out that when safe objectives of the organizer conflicted with production objectives, workers tend to ignore work safety, which led to ineffectiveness of safe incentives. Zhou et al. (2011) concluded that the safe awareness of workers could be improved with a safe environment which has safety regulations, trainings and publicity. Sparer and Dennerlein (2013) collected the safety inspection data of 19 construction projects owned by Harvard University to calculate the frequency and distribution in the incentive plan every month and designed a safety incentive plan to ensure fairness and competitiveness. Ji et al. (2021) researched the tournament incentive mechanism of construction workers' safety behavior with considering multiple heterogeneity and found workers with a risk aversion attitude and a higher level of fairness preference need higher incentive. However, high incentives from managers would only improve workers' safety performance in the short term with its decreased value and shortening the time interval between incentives is more beneficial to promote safe performance (Ghasemi et al., 2015). Generally speaking, safety management has a significant impact on the unsafe behavior of workers, and the incentive and punishment mechanism is an important stimulus. However, most of the above literatures analyzed the impact mechanism of safety management measures such as incentives, and most of them were qualitative. There is a lack of theoretical research on workers' behavioral decisions under incentive or punishment safety management strategies.

While the above studies confirm the necessity of extended and wellplanned management measures to improve safety, the responses of workers to these management strategies, including their learning and evolutionary behaviors should not be ignored. According to social learning theories, human learning includes two types of behavior, one is personal learning, which changes behavior through constant trial and error, and the other one is social learning, which alters its own behavior by imitating others (Mesoudi, 2011).

This study employs the evolutionary game method in order to analyze the evolutionary behavior caused by changing one's own strategies due to learning. The game theory, originated from the theory of biological evolution, is a group behavior analysis theory based on the framework of bounded rationality. It can be described as a mathematical model of strategic interaction between independent subjects. Effective solutions are likely to be determined through the simulation and analysis of the situation in the model so as to provide participants with the best decision-making strategy (Brickley et al., 2000). Smith and Price (1973) established the evolutionary stability strategy, which reflected the dynamic balance of the game behavior of bounded rational groups more accurately. Nowadays, scholars have adopted the game theory in many fields to study the decision-making and evolutionary problems including interactive strategies (Ji et al., 2021). For example, Meng et al. (2021) analyzed the game behaviors of the government and contractors under four different bonus and penalty strategies in green building projects, and then discovered that dynamic incentives as well as static punishments were the best strategies for contractors to promote projects. Song et al. (2021) suggested that the interests and costs of users should be taken into account in the decision-making stage after analyzing the tripartite game among users, public sectors and private sectors in user paid PPP projects. Lv et al. (2021) investigated the evolution of concession renegotiation behaviors when the actual flow in PPP transportation project was inconsistent with the expected flow through evolutionary game, which provides decision support for the governance of concession renegotiation behaviors in PPP projects. Loghman et al. (2022) established a mixed-integer programming model consisting of game theory and project schedule to reduce the duration of grand infrastructure projects with a minimum increase in cost. Luo et al. (2021) used a cooperative game theory to determine the optimal distributed photovoltaic system operation strategy with a benefit analysis to promote the low-carbon economy. Fang and Ding (2009) established a game model among miners, regulatory authorities and coal factories with the analysis of the related safety management, and then made suggestions about the safety management. Yang and Wang (2022) built a three-party game model by investigating the relationships among construction supervision units, construction enterprises and workers, and then concluded that increasing the punishment for violations of workers as well as increasing the benefits and incentives for operations in accordance with regulations would promote behaviors of workers. Wang et al. (2016) employed the game theory to research on the willingness of workers in specific groups to participate in the safety management and found that the willingness of workers to participate in the safety management could be realized by improving workers' rational cognitions of the investment benefits from safe behaviors. These studies have proved that the evolutionary game can be effective in studying the strategic interaction between two parties.

This paper considers that contractors have established both incentive and penalty measures. In view of the long-term nature of safety management, contractors and workers will adjust their strategies through learning by comparing incentive and penalty measures during the progress of the project. This paper aims to: (1) establish an evolutionary game model to investigate the relationship between contractors' active and passive supervision strategies and workers' safe and unsafe behaviors; (2) examine the evolutionary stability of strategies, which represent the equilibrium state of the system after a long interaction from both contractors' and workers' point of view. (3) demonstrate the effectiveness of the model in finding optimal parameter settings in a few scenarios. This paper is organized by the below structure. This paper begins with assumptions about the income of contractors and workers and then forms the payment matrix of both parties. Based on the research method of the evolutionary game, the evolutionary stability of the strategies from both sides is investigated. This paper then develops the scenarios under diverse conditions according to the analysis results of the evolutionary stability for classifications, and the management significance of the scenarios is discussed. With the sensitivity analysis of parameters, the impacts of initial strategies and parameters on evolutionary directions are analyzed respectively. Finally, a summary is given on account of the results of this study.

## Model Establishment and Analysis

### Assumptions and Parameters

In this paper, there are two parties, contractors and construction workers, in the safety management process. Contractors can adopt active supervision or passive supervision. When contractors adopt active supervision to reduce safe incidents, more resources are devoted to the safety management, such as worker safety training and security equipment. The construction workers can adopt safe behaviors or unsafe behaviors. When workers adopt safe behaviors, workers need to improve security awareness through safety training and cooperate with the contractor's regulations to adopt safe measures positively. It is assumed that the occurrence of risk events has a certain probability and is directly related to unsafe behaviors of construction workers. When construction workers have unsafe behaviors, risk events have great potential to happen. Contractors need to establish incentive and penalty measures for behavioral change of workers. Contractors will be punished by government for workers' unsafe behaviors which are periodically inspected. As a result, contractors have intrinsic motivations to improve their corporate reputations.

It is further assumed that if a risk event occurs, there will be a loss of “*L.”* The occurrence of the risk events is related to the behavior strategies adopted by the workers. When workers demonstrate safe behaviors, the probability of occurrence is “*p*_1_*.”* When workers demonstrate unsafe behaviors, the probability of occurrence is “*p*_2_*,”* and obviously in near all construction projects, “*p*_1_” < “*p*_2_*.”* Contractors and construction workers have different perception related to the coefficients of loss, which are “α” and “β” respectively. When contractors adopt a passive supervision strategy, the behavioral characteristics of workers can be observed under a certain incentive “*R*” and a punishment “*G.”* On the other hand, if an active supervision strategy is selected, contractors pay the supervision cost of “*C*_1_” and workers will pay the cost of “*C*_2_” to correct their behaviors. When both parties adopt active strategies at the same time, contractors will receive the reputational income “*F*” for enhanced corporate reputations and social responsibilities. If unsafe behaviors of workers are identified by the government, contractors will receive corresponding penalties. The severity of the penalty varies, depending on whether the contractors take an active supervision strategy or not. If contractors take active supervision strategy, the penalty is “*Q*_1_*.”* Otherwise, the penalty is “*Q*_2_*,”* and obviously, “*Q*_1_” < “*Q*_2_*.”* The model parameters and variables are listed in Table 1.

**Table 1:** Main parameters and descriptions.

**Parameters**	**Descriptions**
*L*	The loss due to risk events
*p*_1_	Probability of risk events when construction workers take safe behavior strategy
*p*_2_	Probability of risk events when construction workers take unsafe behavior strategy
α	Contractors' perception on the coefficient of risk loss
β	Workers' perception on the coefficient of risk loss
*R*	If workers take safe behavior strategy, contractors give incentive to workers
*G*	If workers take unsafe behavior strategy, contractors impose a penalty on workers
*C*_1_	The supervision cost of contractors under active supervision
*C*_2_	The cost of workers to take safe behaviors under active supervision
*F*	Reputation income of contractors
*Q*_1_	The government imposes fine on contractors because of workers' unsafe behaviors under active supervision
*Q*_2_	The government imposes fine on contractors because of the workers' unsafe behaviors under passive supervision

The game payoffs matrix of contractors and construction workers can therefore be constructed, as shown in Table 2.

**Table 2:** The payoffs matrix of contractors and construction workers.

		**Construction workers**	
		**Safe behavior**	**Unsafe behavior**
Contractors	Active	−α*p*_1_*L* − *R* − *C*_1_ + *F*	−α*p*_2_*L* + *G* − *C*_1_ − *Q*_1_
	supervision	−β*p*_1_*L* + *R* − *C*_2_	−β*p*_2_*L* − *G*
	Passive	−α*p*_1_*L*	−α*p*_2_*L* − *Q*_2_
	supervision	−β*p*_1_*L* − *C*_2_	−β*p*_2_*L*

### Solution and Analysis of Evolutionary Stability Strategy

Assuming that the probability of contractors adopting an active supervision strategy is *x*, therefore the probability of adopting a passive supervision strategy is 1 − *x*. The probability of construction workers adopting a safe behavior strategy is *y*, and the probability of adopting an unsafe behavior strategy is 1 − *y*.

The expected revenue under the condition of an active supervision or a passive supervision are *w*_1*p*_, *w*_1*n*_ and its mean average for contractors is *w*_1_. They can be calculated as follows:


(1) $$\begin{array}{l} w_{1p}=y\left(-\alpha p_{1}L-R-C_{1}+F\right)\\ +\left(1-y\right)\left(-\alpha p_{2}L+G-C_{1}-Q_{1}\right) \end{array}$$

(2) $$\begin{array}{l} w_{1n}=y\left(-\alpha p_{1}L\right)+\left(1-y\right)\left(-\alpha p_{2}L-Q_{2}\right) \end{array}$$

(3) $$\begin{array}{l} w_{1}=xw_{1p}+(1-x)w_{1n}=x(y(-R-G+Q_{1}-Q_{2}+F)\\ +G-C_{1}-Q_{1}+Q_{2})+y(\alpha (p_{2}-p_{1})L+Q_{2})-\alpha p_{2}L-Q_{2} \end{array}$$

The expected revenue under the condition of safe behaviors and unsafe behaviors are *w*_2*s*_,*w*_2*u*_ and its mean average for construction workers is *w*_2_. They can be calculated as follows:


(4) $$\begin{array}{l} w_{2s}=x\left(-\beta p_{1}L+R-C_{2}\right)\\ +\left(1-x\right)\left(-\beta p_{1}L-C_{2}\right) \end{array}$$

(5) $$\begin{array}{l} w_{2u}=x\left(-\beta p_{2}L-G\right)\\ +\left(1-x\right)\left(-\beta p_{2}L\right) \end{array}$$

(6) $$\begin{array}{l} w_{2}=yw_{2s}+(1-y)w_{2u}=y(x(R+G)\\ +\beta (p_{2}-p_{1})L-C_{2})+x(-G)-\beta p_{2}L \end{array}$$

According to the theory of evolutionary game (Swinkels, 1993), the replicated dynamic equations of contractors and construction workers can be obtained as follow. For the convenience of calculation, let *Q*_2_ − *Q*_1_ = Δ*Q*.


(7) $$\begin{array}{l} F\left(x\right)=\frac{dx}{dt}=x\left(w_{1p}-w_{1}\right)\\ =x\left(1-x\right)\left[y\left(-R-G-\Delta Q+F\right)+G-C_{1}+\Delta Q\right] \end{array}$$

(8) $$\begin{array}{l} G(y)=\frac{dy}{dt}=y(w_{2s}-w_{2})\\ =y(1-y)[x(R+G)+\beta (p_{2}-p_{1})L-C_{2}] \end{array}$$

The corresponding Jacobi matrix is:


(9) $$\begin{array}{l} J=\left[\begin{array}{c} a_{11}\;a_{12}\\ a_{21}\;a_{22} \end{array}\right] \end{array}$$

Corresponding:


(10) $$\begin{array}{l} a_{11}=\left(1-2x\right)\left[y\left(-R-G-\Delta Q+F\right)+G-C_{1}+\Delta Q\right] \end{array}$$

(11) $$\begin{array}{l} a_{12}=x\left(1-x\right)\left(-R-G-\Delta Q+F\right) \end{array}$$

(12) $$\begin{array}{l} a_{21}=y\left(1-y\right)\left(R+G\right) \end{array}$$

(13) $$\begin{array}{l} a_{22}=\left(1-2y\right)\left[x\left(R+G\right)+\beta \left(p_{2}-p_{1}\right)L-C_{2}\right] \end{array}$$

The trace of the Jacobi matrix can be obtained by solving (14):


(14) $$\begin{array}{l} Tr\;J=(1-2x)[y(F-R-G-Q)+G-C_{1}+Q]+(1-2y)\\ \;[x(R+G)+\beta (p_{2}-p_{1})L-C_{2}] \end{array}$$

The determinant of Jacobi matrix can be obtained by solving (15):


(15) $$\begin{array}{l} Det\;J=(1-2x)[y(F-R-G-\Delta Q)\\ +G-C_{1}+\Delta Q](1-2y)[x(R+G)+\beta (p_{2}-p_{1})L-C_{2}]\\ -x(1-x)(F-R-G-\Delta Q)y(1-y)(R+G) \end{array}$$

According to the replicated dynamic equations of contractors and construction workers, there are five equilibrium points. These five equilibrium points are (0,0), (0,1), (1,0), (1,1) and (x^*^, y^*^), with:


(16) $$\begin{array}{l} x^{*}=\frac{C_{2}-\beta (p_{2}-p_{1})L}{R+G} \end{array}$$

(17) $$\begin{array}{l} y^{*}=\frac{C_{1}-G-\Delta Q}{F-R-G-\Delta Q} \end{array}$$

Among these five equilibrium points, (0,0) represents that the contractors take the passive supervision while the construction workers take the unsafe behaviors. (0,1) represents that the contractors take passive supervision while the construction worker adopts safe behaviors. (1,0) represents that contractors take the active supervision while construction workers take unsafe behaviors. (1,1) represents that contractors take the active supervision while the construction workers take safe behaviors. And (x^*^, y^*^) represents that both contractors and construction workers take the mixed strategies, that is, both positive and negative strategies exist. The determinant and trace of the Jacobi matrix at different equilibrium point are shown in Table 3.

**Table 3:** The determinant and trace of the Jacobi matrix at different equilibrium point.

**Equilibrium point**	***Det J***	***Tr J***
(0,0)	(*G* − *C*_1_ + Δ*Q*)*[β(*p*_2_ − *p*_1_)*L* − *C*_2_]	*G* − *C*_1_ + Δ*Q* + β(*p*_2_ − *p*_1_)*L* − *C*_2_
(1,0)	−(*G* − *C*_1_ + Δ*Q*)*[(*R* + *G*) + β(*p*_2_ − *p*_1_)*L* − *C*_2_]	*C*_1_ − Δ*Q* + *R* + β(*p*_2_ − *p*_1_)*L* − *C*_2_
(0,1)	(*F* − *R* − *C*_1_)* − [β(*p*_2_ − *p*_1_)*L* − *C*_2_]	(*F* − *R* − *C*_1_) − [β(*p*_2_ − *p*_1_)*L* − *C*_2_]
(1,1)	(*F* − *R* − *C*_1_)[(*R* + *G*) + β(*p*_2_ − *p*_1_)*L* − *C*_2_]	−*F* + *C*_1_ − *G* − β(*p*_2_ − *p*_1_)*L* + *C*_2_
(x^*^, y^*^)		*0*

Under the evolutionarily stable strategy, neither contractor nor construction workers can achieve greater benefits by changing their own strategies, thus resulting in a stable state of strategies for both parties. According to evolutionary stability, the evolutionary states of each point are saddle point, instability points and evolutionary stable strategy (ESS) respectively when the value and trace of each equilibrium point are (−, *N*), (+, +) and (+, −). According to the assumptions in this paper, different parameter ranges are calculated separately, and 12 evolutionary stability scenarios are obtained and shown in Appendix 1.

## Scenario Analysis

**Figure 1:**
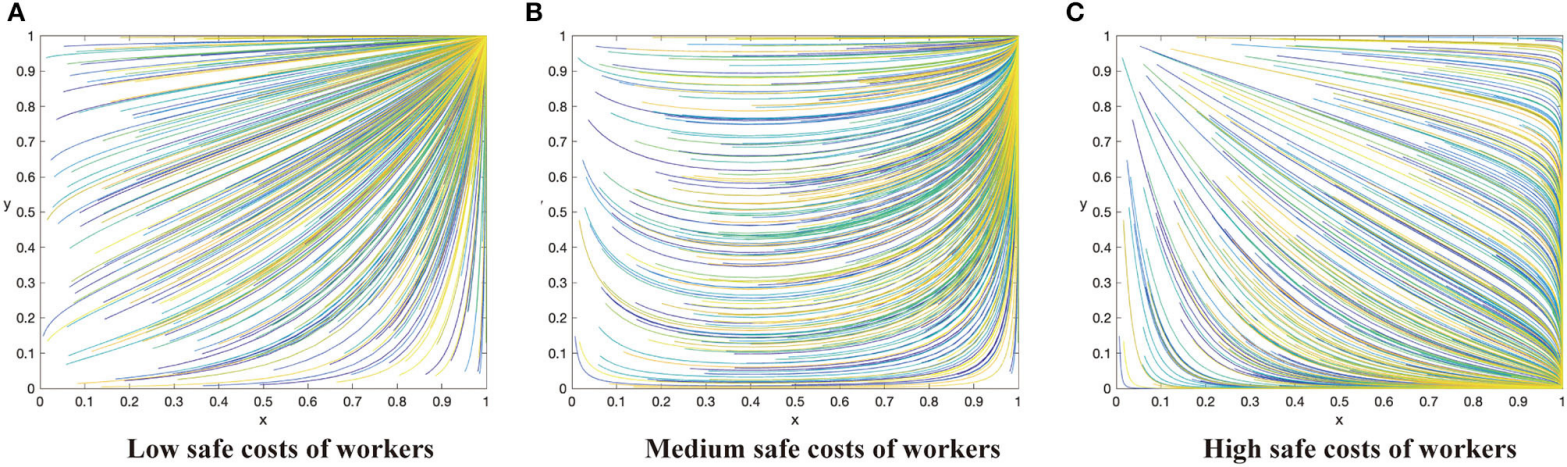
The dynamic evolutionary path of the game in category 1, including low safety costs to workers **(A)**, high safety costs to workers **(C)**, and medium safety costs to workers **(B)**.

**Figure 2:**
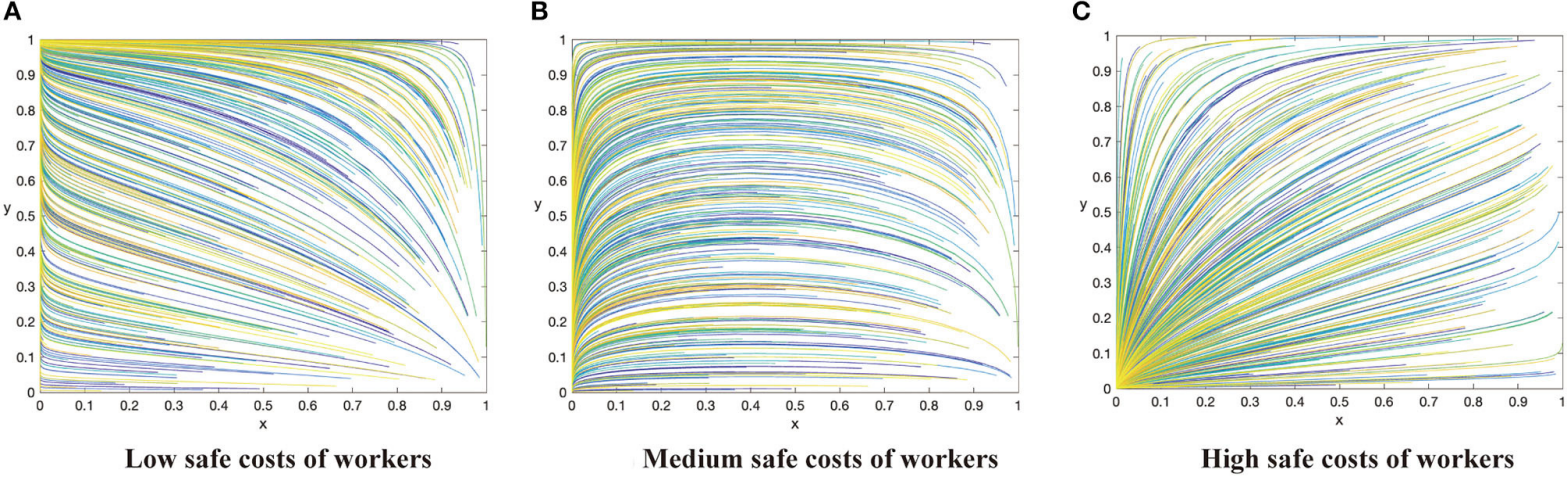
The dynamic evolutionary path of the game in category 2, including low safety costs to workers **(A)**, high safety costs to workers **(C)**, and medium safety costs to workers **(B)**.

**Figure 3:**
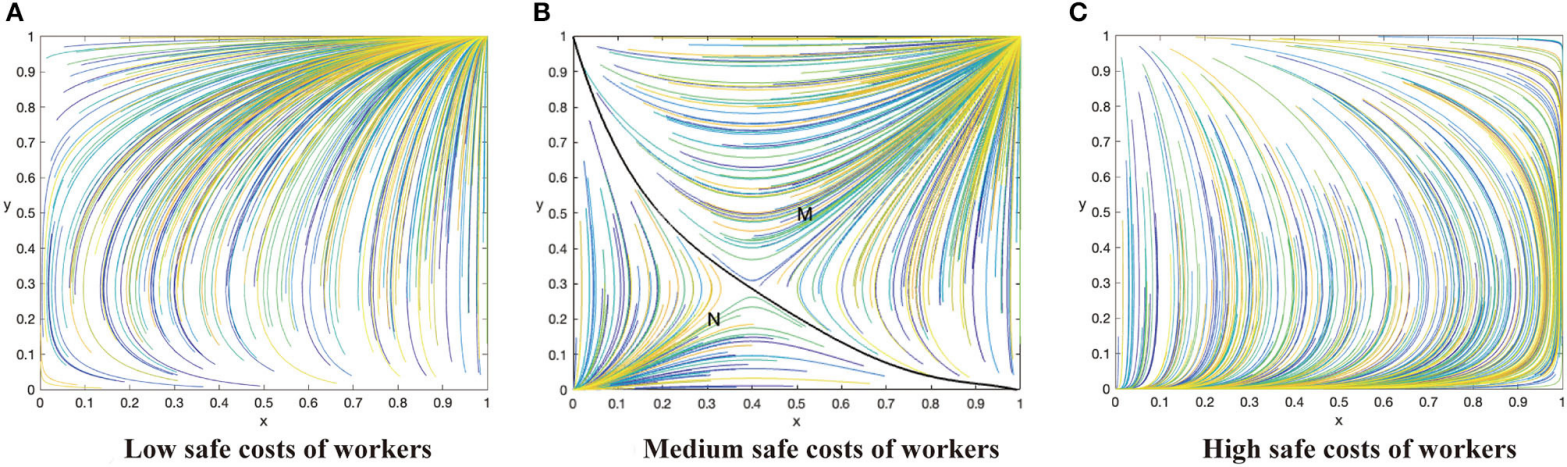
The dynamic evolutionary path of the game in sub-category 3-1, including low safety costs to workers **(A)**, high safety costs to workers **(C)**, and medium safety costs to workers **(B)**.

**Figure 4:**
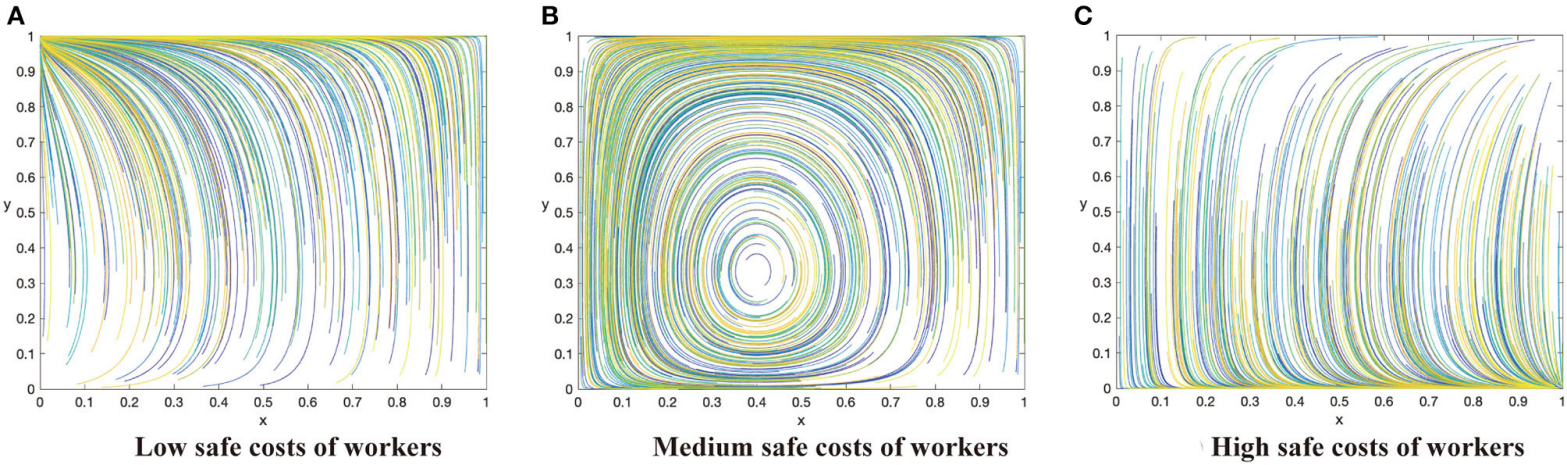
The dynamic evolutionary path of the game in sub-category 3-2, including low safety costs to workers **(A)**, high safety costs to workers **(C)**, and medium safety costs to workers **(B)**.

Based on the above analysis process, there are 12 evolutionary stable scenarios for contractors and workers. Considering the fact that supervision cost is the main factor preventing contractors from adopting the active supervision strategies to improve safety performance, this study further categorizes the 12 scenarios into three categories based on the costs to contractors. The first category reflects that supervision cost of contractors is lower than the sum of punishment fees from both workers and the government as well as the differences between potential income brought by reputation and incentive investment, i.e., *C*_1_ < min{*G* + Δ*Q, F* − *R*}. There are three scenarios when the contractors have a strong motivation to take active supervision. By considering the parameters under the corresponding restrictions, the evolution scenarios are shown in Figure 1. The second category reflects that supervision cost of contractors is greater than the sum of punishment fees from both workers and the government as well as the differences between potential income brought by reputation and incentive investment, i.e., *C*_1_ > min{*G* + Δ*Q, F* − *R*}. There are three scenarios when the contractors have to pay a high supervision cost, which are shown in Figure 2. The third category reflects that supervision cost is between the sum of punishment fees from both workers and the government and the differences between potential income brought by reputation and incentive investment, i.e., min{*G* + Δ*Q, F* − *R*} < *C*_1_ < *ma*x{*G* + Δ*Q, F* − *R*}. In this condition, the strategies of contractors are uncertain and it has six scenarios, which is shown in Figures 3, 4.

### Category 1: Supervision Cost of Contractors Is low

Conclusion 1: When the supervision cost is low, that is *C*_1_ < *min*{*G* + Δ*Q, F* − *R*}, there will be three evolutionary situations and two evolutionary stable states, depending on the cost of construction workers to take safe behaviors. When *C*_2_ > (*R* + *G*) + β(*p*_2_ − *p*_1_)*L*, the ESS of the system is (1,0). When *C*_2_ < (*R* + *G*) + β(*p*_2_ − *p*_1_)*L*, the evolution trend of the system is slightly different in two cases, but it finally levels off at (1,1). These three scenarios are shown in Figures 1A–C respectively. The horizontal axis represents the percentage of contractors using the active supervision strategy and the vertical axis represents the percentage of construction workers taking safe behaviors in Figure 1, the same in Figures 2–4.

Managerial implications of Conclusion 1. Conclusion 1 demonstrates a situation when the supervision cost is lower than the sum of punishment fees from both workers and the government as well as the differences between potential income brought by enhanced reputation and incentive investment. Under this situation, the system will evolve in a positive direction when the cost of workers to take safe behaviors is relatively low. In the two sub-categories above (Figures 1A,B), construction workers will evolve to take safe behaviors if the cost of workers is less than β(*p*_2_ − *p*_1_)*L*, which is the utility improvement brought by safe behaviors. When the populations of contractors adopting positive supervision strategy is low, construction workers evolve in the direction of taking unsafe behaviors. On the other hand, with the number of contractors adopting positive supervision strategy increases, the evolution direction of construction workers is to take safe behaviors, and finally the system evolves in a positive direction. The groups of contractors and workers tend to achieve stability at (1,1) respectively in the end. If the effort cost of workers further increases, the effort strategy employed by construction workers will not pay off. Under this situation, although the contractors evolve in the direction of active supervision (i.e., by paying more in active supervision), they exert not enough influence on construction workers to change their behaviors (i.e., evolution trend). Both parties achieve a stable state in (1,0) finally (Figure 1C).

### Category 2: Supervision Cost of Contractors Is High

Conclusion 2: When the supervision cost is high, that is *C*_1_ > *max*{*G* + Δ*Q, F* − *R*}, there will be three evolutionary situations and two evolutionary stable states based on the value of safety costs to construction workers. When *C*_2_ < β(*p*_2_ − *p*_1_)*L*, the ESS of the system is (0,1). When *C*_2_ > β(*p*_2_ − *p*_1_)*L*, the evolution trend of the system is slightly different in two cases, but it is stable at (0,0) finally. All these are shown in Figures 2A–C respectively.

Managerial implications of Conclusion 2. Conclusion 2 demonstrates a situation when the supervision cost is higher than the sum of punishment fees from both workers and the government as well as the differences between potential income brought by enhanced reputation and incentive investment. Under this situation, the system will evolve in a negative direction when the cost of workers is relatively high. In the two sub-categories above (Figures 2B,C), construction workers will evolve to take unsafe behaviors if the cost of workers is greater than the utility improvement brought by safe behaviors. With the number of contractors adopting positive supervision strategy decreasing, the evolution direction of construction worker populations is to take safe behaviors, and finally the system evolves in a negative direction. In the two sub-categories above (Figures 2B,C), contractors and workers will finally achieve stability at (0,0). If the cost to construction workers is further reduced, and the utility improvement brought by safe behaviors can offset the effort cost completely, both sides achieve a stable state at (0,1) (Figure 2A).

### Category 3: Supervision Cost of Contractors Is in the Middle

When the supervision cost is in the middle, the evolution direction of the system will be divided into two scenarios. The first sub situation is *F* > *G* + Δ*Q* + *R* where the corporate social responsibility perception is relatively larger. Enterprises are willing to establish a positive corporate image and avoid workplace casualties as much as possible, which is common for large enterprises and state-owned enterprises. The other scenario is *F* < *G* + Δ*Q* + *R*, when the benefit of building a reputation is relatively lower.

#### Sub-category 3-1: The Benefits of Corporate Social Responsibility Perceptions Are of Great Value

Conclusion 3: When the supervision cost is in the middle state, that is *G* + Δ*Q* < *C*_1_ < *F* − *R*, there will be three evolutionary situations and three evolutionary stable states based on the value of safety costs to workers. When *C*_2_ < β(*p*_2_ − *p*_1_)*L*, the ESS of the system is (1,1). When β(*p*_2_ − *p*_1_)*L* < *C*_2_ < (*R* + *G*) + β(*p*_2_ − *p*_1_)*L*, the system has two ESS (0,0) and (1,1). When *C*_2_(*R* + *G*) + β(*p*_2_ − *p*_1_)*L*, the ESS of the system is (0,0). All these are shown in Figure 3.

Managerial implications of Conclusion 3. Conclusion 3 demonstrates a situation when the supervision cost is in the middle between the potential benefit and the cost of taking active supervision. The system has a relatively diverse evolutionary stable state. The system will be stable at (0,0) when the cost of construction workers is greater than the utility improvement combined with incentives and punishments brought by taking safe behaviors (Figure 3C). As the costs to workers increase, the system will eventually evolve in the negative direction although the evolution trend of contractors is positive for a time. If the cost to construction workers decreases slightly, but is not lower than the utility improvement brought by taking safe actions, two ESSs will appear in the system. At this time, the evolution direction depends on the initial state of the system. The system will evolve toward (1,1) if the mixed strategies of the contractors and workers are transferred to area M (the bottom left area of Figure 3B), while the system will evolve toward (0,0) if the mixed strategies of contractors and workers are transferred to area N (the upper right area of Figure 3B). A mixed strategy is one where contractors and construction workers will randomly choose their strategies with some probability. Whether contractors and works have a positive attitude at the beginning is very significant to determine the evolution direction of the system, so contractors can strengthen mutual trust and cooperation to promote the evolution positively. The area M represents the probability that the two sides will active strategies which is related to (x^*^, y^*^). When the cost to workers is lower than the utility improvement brought by safe behaviors, the effort cost can be offset completely, therefore the system will evolve toward (1,1). With the number of construction workers taking safe behaviors increases, the evolution direction of groups of contractors is to take active supervision. Finally, both parties obtain a stable state at (1,1) (Figure 3A).

#### Sub-category 3-2: The Benefits of Corporate Social Responsibility Perceptions Are of Small Value

Conclusion 4: When the supervision cost is in the middle state, that is *G* + Δ*Q* < *C*_1_ < *F* − *R*, there will e three evolutionary situations and three evolutionary stable states based on the value of safety cost to construction workers. When *C*_2_ < β(*p*_2_ − *p*_1_)*L*, the ESS of the system is (0,1). When β(*p*_2_ − *p*_1_)*L* < *C*_2_ < (*R* + *G*) + β(*p*_2_ − *p*_1_)*L*, there will be only hybrid strategies and no evolutionary stable point ESS. When *C*_2_(*R* + *G*) + β(*p*_2_ − *p*_1_)*L*, the ESS of the system is (1,0). The three scenarios are shown in Figures 4A–C respectively.

Managerial implications of Conclusion 4. Conclusion 4 demonstrates a situation when the supervision cost is in the middle between the potential benefit and the cost of taking active supervision, which is similar to Conclusion 3, but in this case the potential benefits of improved reputation is low. The system will be stable at (1,0) when the cost of workers is greater than the utility improvement combined with incentives and punishments brought by safe behaviors (Figure 4C). Contractors will involve in the direction of passive supervision. However, with the number of workers taking unsafe behaviors increases, contractors begin to strengthen supervision gradually with the intention of strengthening the punishment of workers and reducing the penalty from the government. If the cost to workers is lower, but is still higher than the utility improvement brought by safe behaviors, the system will be unstable (Figure 4B). If the cost of workers is lower than the utility improvement brought by safe behaviors, the improved effectiveness can offset the cost completely, therefore the system will evolve toward (0,1). With the number of workers with safe behaviors increasing, the evolution direction of contractors is to take active supervision reversely and finally the evolution direction of contractors will turn to passive supervision. Both parties will obtain a stable state at (0,1) in order to reduce the cost of supervision (Figure 4A).

## Sensitivity Analysis

### The Impact of Initial Value

**Figure 5:**
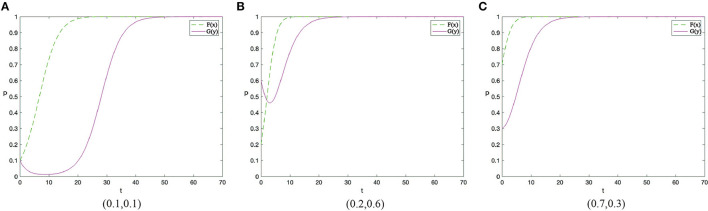
The evolutionary trajectories of (0.1,0.1) **(A)**, (0.2,0.6) **(B)**, and (0.7,0.3) **(C)** in scenario 1.

It is necessary to examine the impact of the initial state (*x, y*) on the final evolutionary stability. The evolution paths under two different scenarios are tested, as shown in Tables 4, 5. In scenario 1, contractors have low supervision cost and construction workers have the medium safety cost. In scenario 2, contractors have the medium supervision cost but the workers' safety cost is the same with scenario 1. Both scenarios are common in engineering field. In scenario 1, the initial state point (0.1, 0.1), when few contractors tend to take active supervision and few workers take safe behaviors, is tested. The results are shown in the Figure 5A. The evolutionary trajectories increase gradually to ESS (1,1). The initial state (0.2, 0.6) and (0.7, 0.3) are tested as well. The results are shown in Figures 5B,C. From Figures 5A–C, we can find that: (1) when the value of parameters keep constant, the initial state (*x, y*) does not affect the final ESS; (2) the difference of the three figures is the time reaching ESS (1,1), which is influenced by the initial state.

**Table 4:** The parameter settings of scenario 1.

**Item**	**F**	**C_1_**	**C_2_**	**R**	**G**	**α**	**β**	**p_1_**	**p_2_**	**L**	**Q_1_**	**Q_2_**
Value	4.5	3.0	1.0	0.5	0.3	0.6	0.4	0.4	0.8	3.0	1.5	4.5

**Table 5:** The parameter settings of scenario 2.

**Item**	**F**	**C_1_**	**C_2_**	**R**	**G**	**α**	**β**	**p_1_**	**p_2_**	**L**	**Q_1_**	**Q_2_**
Value	4.5	3.5	1.0	0.5	0.3	0.6	0.4	0.4	0.8	3.0	1.5	4.5

**Figure 6:**
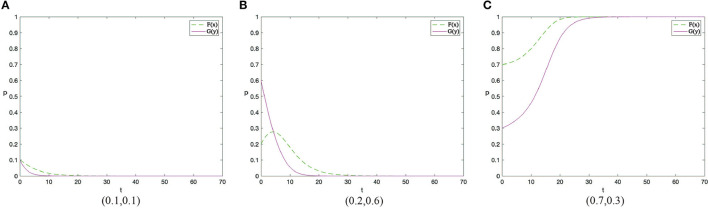
The evolutionary trajectories of (0.1,0.1) **(A)**, (0.2,0.6) **(B)** and (0.7,0.3) **(C)** in scenario 2.

In scenario 2, the evolution paths under the initial state points (0.1,0.1), (0.2,0.6), and (0.7,0.3) are also tested. The results are shown in Figures 6A–C respectively. By comparing these three figures, we can see that (1) the initial values of (*x, y*) will affect the final ESS under this parameter condition; (2) the time reaching ESS will be affected by the initial values of (*x, y*) as well.

### Single-Factor Sensitivity Analysis

Finally, sensitivity analysis on individual factors is also conducted. The value of an individual factor is adjusted while other variables are kept consistent (as shown in Table 6) to observe the impact of this factor on the evolution, so as to help managers to adjust behavior strategies more targeted. This analysis considers reputation incomes for contractors “*F*,” rewards for workers' safe behaviors “*R*, ” and penalty costs for workers' unsafe behaviors on the evolution results “*G*” which have a great impact on behavior strategies of contractors and workers. The value setting of other variables represents a scenario when contractors have low supervision cost and construction workers have high safety cost which is a common phenomenon in practical situations.

**Table 6:** The parameter settings of single-factor sensitivity analysis.

**Item**	**C_1_**	**C_2_**	**α**	**β**	**p_1_**	**p_2_**	**L**	**Q_1_**	**Q_2_**
Value	3.0	1.4	0.6	0.4	0.4	0.8	3.0	1.5	4.5

**Figure 7:**
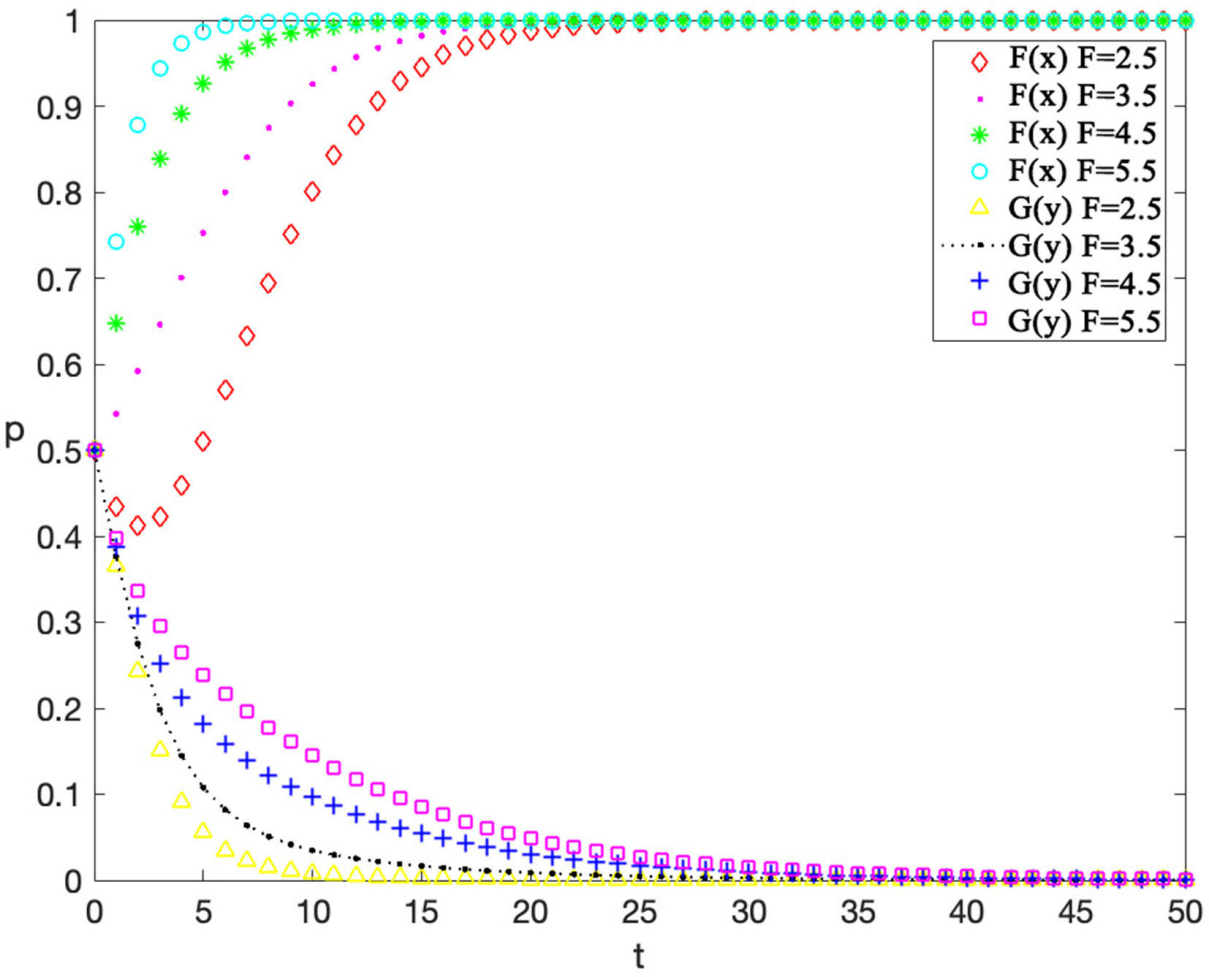
The evolutionary trajectories influenced by F.

The sensitivity analysis of “*F”* is shown in Figure 7 and the values of “*R*” and “*G*” are 0.5 and 0.3 respectively. According to this figure, changing reputation incomes does not affect the final evolution result. However, it does influence the speed of evolution. With the increasing of reputational benefit, contractors are more likely to adopt active supervision, thus leading to a reduction in the probability of the workers' unsafe behaviors. This phenomenon suggests that the behavior of contractors and workers can be effectively guided and corrected by enhancing the contractor's perception of reputational income.

**Figure 8:**
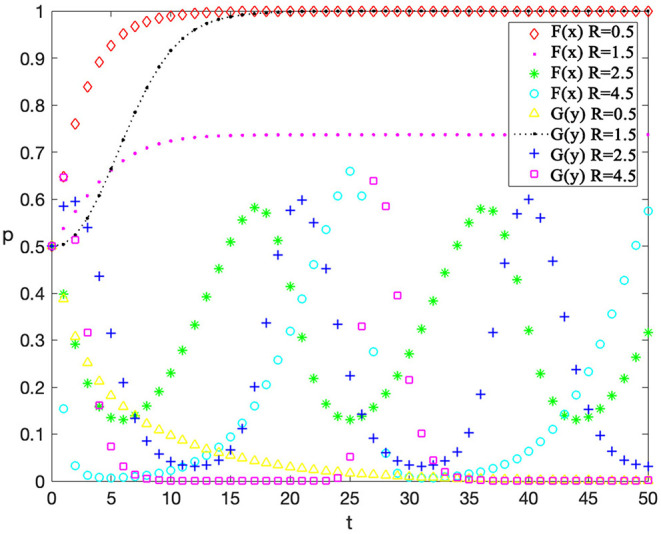
The evolutionary trajectories influenced by R.

The sensitivity analysis of incentive “*R”* for worker's safe behavior is shown in Figure 8 and the values of “*F*” and “*G*” are 4.5 and 0.3 respectively. It can be found that when contractors provide workers with more incentives, contractors take a lower active supervision and workers tend to take safe behaviors as the equilibrium point changes from (1,0) to (1,1). When “*R”* continues to increase, the strategies of both parties are then unstable. This phenomenon demonstrates that the incentive provided by contractors to workers should be established reasonably so that it can help increase the probability of workers to take safe behaviors as well as help contractors avoid the loss of taking excessive active supervision.

**Figure 9:**
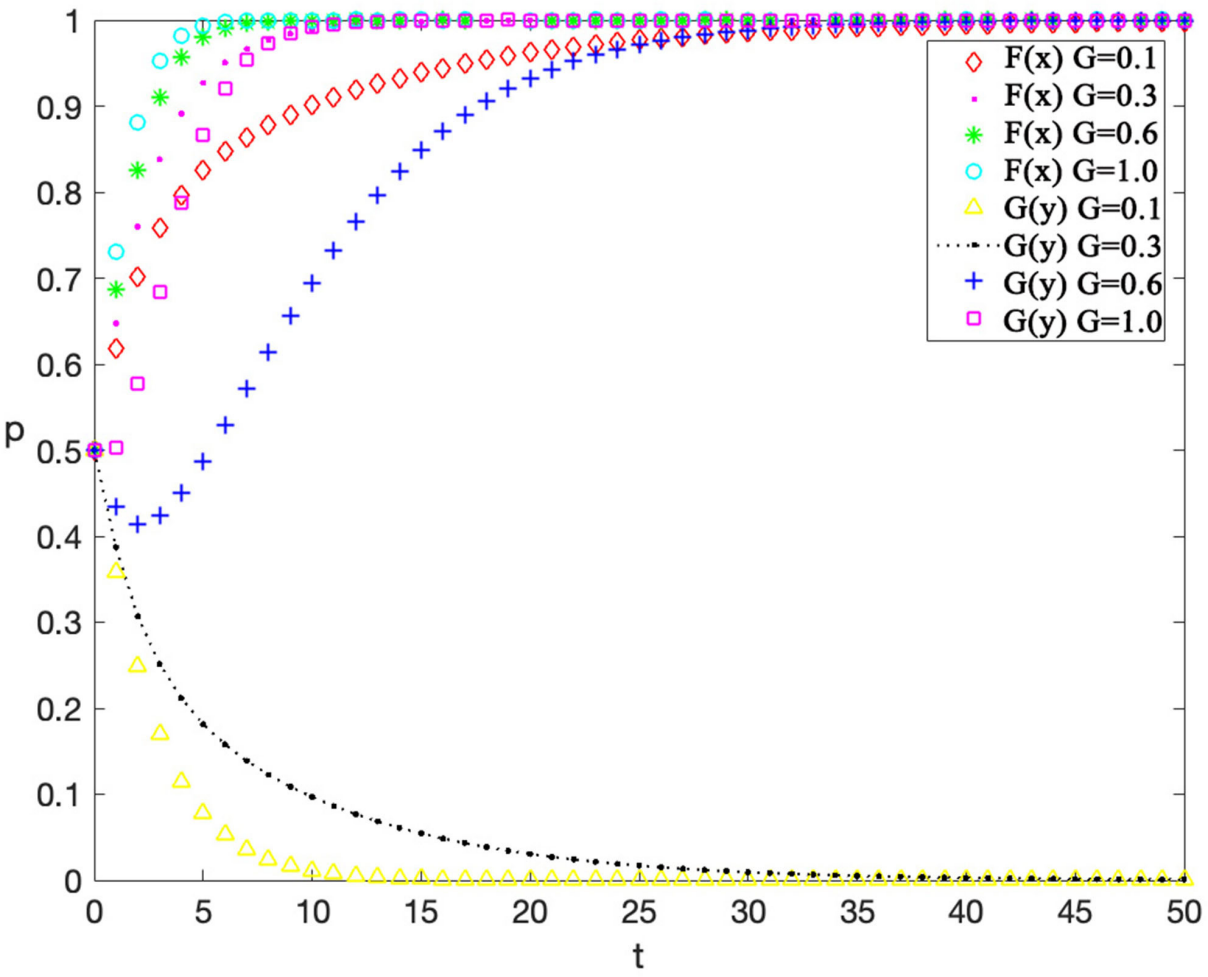
The evolutionary trajectories influenced by G.

The sensitivity analysis of the contractors' penalty “*G”* for unsafe behaviors is shown in Figure 9 and the values of “*F*” and “*R*” are 4.5 and 0.5 respectively. It can be found that as the penalty costs for unsafe behaviors increase, contractors are more likely to adopt an active supervision strategy, thus reducing the possibility of unsafe behaviors by workers. Finally, workers will take passive safe behaviors because they want to avoid unbearable penalties. Therefore, contractors will have sufficient profits to take active supervision. This phenomenon suggests that the probability of unsafe behavior of workers can be reduced by increasing the penalty.

## Conclusion

In this paper, we construct a game model between contractors and workers, and then focus on comparing the evolution process of supervision behaviors from contractors and safe or unsafe behaviors from workers under different incentive and penalty mechanisms. The impact of the initial state and parameters on the behavior strategies of both parties is further investigated through simulation.

This has the following contributions to new knowledge related to establishing best strategies to improve safety performance of contractors and workers:

1. The government and contractors should establish a reasonable incentive and penalty mechanism as a reasonable benefit allocation is crucial to enhance cooperation (Li et al., 2018). The analysis of evolutionary stability and sensitivity shows that the settings of incentive and punishment variables can impact the evolution direction and speed of strategies from contractors and workers. Other parameters (e.g., reputational benefit) are greatly affected by external factors, incentive and penalty mechanisms are completely dominated by contractors. Contractors and workers can adopt more positive strategies with appropriative incentive and penalty mechanisms.
2. Mutual trust between contractors and workers should be strengthened as mutual trust can keep cooperative evolutionary direction and cooperative stability (Xue et al., 2010). The initial perceptions of both sides have a decisive impact on the evolution direction. Both parties are encouraged to strengthen communications with increased mutual trust as the results show that if the number of contractors and workers taking positive strategies increases at the beginning, a positive evolutionary stability can be obtained faster. In addition, more positive perceptions of both sides at the initial stage will lead to a positive evolution more quickly, which reduces the trial and error cost of both sides.
3. Contractors are encouraged to raise the awareness of social responsibilities with reduced supervision costs, and improve the safety communication, education and publicity of construction workers. Strengthening safety governance has urgently become social responsibility that can't be shifted by contractors and government supervision departments (Zhang, 2009). The awareness of social responsibilities can be raised through a few strategies like the construction of the social credit system. Meanwhile, the supervision cost is likely to be reduced through the application of intelligent monitoring and warning technologies. Safety communication, education and publicity will help construction workers determine the suitable costs. These, if combined with an appropriate incentive and penalty mechanism, can help enhance the safety performance for both contractors and construction workers.

## Data Availability Statement

The original contributions presented in the study are included in the article/Supplementary Material, further inquiries can be directed to the corresponding author/s.

## Author Contributions

JZ contributed to conception, modeling, experiment, and writing. CZ contributed to the experiment and writing. SW contributed to the writing and checking. JY and QL contributed to review, editing, and funding acquisition. All authors have read and agreed to the published version of the manuscript.

## Funding

This work was supported by the National Natural Science Foundation of China (Nos. 72101055, 51978164, 72071096, and 71871113), the China Postdoctoral Science Foundation (2021M690607), Social Science Fund of Jiangsu Province (20GLC019), and the Fundamental Research Funds for the Central Universities (2242021R20026).

## Conflict of Interest

The authors declare that the research was conducted in the absence of any commercial or financial relationships that could be construed as a potential conflict of interest.

## Publisher's Note

All claims expressed in this article are solely those of the authors and do not necessarily represent those of their affiliated organizations, or those of the publisher, the editors and the reviewers. Any product that may be evaluated in this article, or claim that may be made by its manufacturer, is not guaranteed or endorsed by the publisher.
