# Supplementary material for: Evolutionary Game Analysis of Construction Workers' Unsafe Behaviors Based on Incentive and Punishment Mechanisms
Source: Front Psychol. 2022 May 24;13:907382. doi: 10.3389/fpsyg.2022.907382 (PMC9172908; doi:10.3389/fpsyg.2022.907382)
Supplement: Supplementary file 1 [file Table_1.docx]

Supplementary Material

# Supplementary Figures and Tables

**Appendix 1. Local stability analysis under different scenarios**

| Equilibrium point | $C_{1}<min\{G+\Delta Q, F-R\}$ | | |  | | |  | | | $C_{1}>max\{G+\Delta Q, F-R\}$ | | |  | | |  | | |
| --- | --- | --- | --- | --- | --- | --- | --- | --- | --- | --- | --- | --- | --- | --- | --- | --- | --- | --- |
|  | $\left( R+G \right)+\beta{(p}_{2}-p_{1})L<C_{2}$ | | | ${(p}_{2}-p_{1})L<C_{2}<\left( R+G \right)+\beta{(p}_{2}-p_{1})L$ | | | $C_{2}<\beta{(p}_{2}-p_{1})L$ | | | $\left( R+G \right)+\beta{(p}_{2}-p_{1})L<C_{2}$ | | | $\beta{(p}_{2}-p_{1})L<C_{2}<\left( R+G \right)+\beta{(p}_{2}-p_{1})L$ | | | $C_{2}<\beta{(p}_{2}-p_{1})L$ | | |
|  | Det j | Tr j | state | Det j | Tr j | state | Det j | Tr j | state | Det j | Tr j | state | Det j | Tr j | state | Det j | Tr j | state |
| $（0,0）$ | $-$ | $N$ | Saddle point | $-$ | $N$ | Saddle point | $+$ | $+$ | Instability point | $+$ | $-$ | ESS | $+$ | $-$ | ESS | $-$ | $N$ | Saddle point |
| $（1,0）$ | $+$ | $-$ | ESS | $-$ | $N$ | Saddle point | $-$ | $N$ | Saddle point | $-$ | $N$ | Saddle point | $+$ | $+$ | Instability point | $+$ | $+$ | Instability point |
| $（0,1）$ | $+$ | $+$ | Instability point | $+$ | $+$ | Instability point | $-$ | $N$ | Saddle point | $-$ | $N$ | Saddle point | $-$ | $N$ | Saddle point | $+$ | $-$ | ESS |
| $（1,1）$ | $-$ | $N$ | Saddle point | $+$ | $-$ | ESS | $+$ | $-$ | ESS | $+$ | $+$ | Instability point | $-$ | $N$ | Saddle point | $-$ | $N$ | Saddle point |
| $（x^{*},y^{*}）$ | Meaningless | | | Meaningless | | | Meaningless | | | Meaningless | | | Meaningless | | | Meaningless | | |

| Equilibrium point | $\min\left\{ G+\Delta Q, F-R \right\}<C_{1}<max\{G+\Delta Q, F-R\}$ | | | | | |  | | |  | | |  | | |  | | |
| --- | --- | --- | --- | --- | --- | --- | --- | --- | --- | --- | --- | --- | --- | --- | --- | --- | --- | --- |
|  | $F>R+G+\Delta Q$ | | |  | | |  | | | $F<R+G+\Delta Q$ | | |  | | |  | | |
|  | $\left( R+G \right)+\beta{(p}_{2}-p_{1})L<C_{2}$ | | | $\beta{(p}_{2}-p_{1})L<C_{2}<\left( R+G \right)+\beta{(p}_{2}-p_{1})L$ | | | $C_{2}<\beta{(p}_{2}-p_{1})L$ | | | $\left( R+G \right)+\beta{(p}_{2}-p_{1})L<C_{2}$ | | | $\beta{(p}_{2}-p_{1})L<C_{2}<\left( R+G \right)+\beta{(p}_{2}-p_{1})L$ | | | $C_{2}<\beta{(p}_{2}-p_{1})L$ | | |
|  | Det j | Tr j | state | Det j | Tr j | state | Det j | Tr j | state | Det j | Tr j | state | Det j | Tr j | state | Det j | Tr j | state |
| $（0,0）$ | $+$ | $-$ | ESS | $+$ | $-$ | ESS | $-$ | $N$ | Saddle point | $-$ | $N$ | Saddle point | $-$ | $N$ | Saddle point | $+$ | $+$ | Instability point |
| $（1,0）$ | $-$ | $N$ | Saddle point | $+$ | $+$ | Instability point | $+$ | $+$ | Instability point | $+$ | $-$ | ESS | $-$ | $N$ | Saddle point | $-$ | $N$ | Saddle point |
| $（0,1）$ | $+$ | $+$ | Instability point | $+$ | $+$ | Instability point | $-$ | $N$ | Saddle point | $-$ | $N$ | Saddle point | $-$ | $N$ | Saddle point | $+$ | $-$ | ESS |
| $（1,1）$ | $-$ | $N$ | Saddle point | $+$ | $-$ | ESS | $+$ | $-$ | ESS | $+$ | $+$ | Instability point | $-$ | $N$ | Saddle point | $-$ | $N$ | Saddle point |
| $（x^{*},y^{*}）$ | Meaningless | | | $<0$ | $0$ | Central point | Meaningless | | | Meaningless | | | $>0$ | $0$ | Central point | Meaningless | | |
